# Dependence of Plant Uptake and Diffusion of Polycyclic Aromatic Hydrocarbons on the Leaf Surface Morphology and Micro-structures of Cuticular Waxes

**DOI:** 10.1038/srep46235

**Published:** 2017-04-10

**Authors:** Qingqing Li, Yungui Li, Lizhong Zhu, Baoshan Xing, Baoliang Chen

**Affiliations:** 1 Department of Environmental Science, Zhejiang University, Hangzhou 310058, China; 2 Zhejiang Provincial Key Laboratory of Organic Pollution Process and Control, Hangzhou 310058, China; 3 Stockbridge School of Agriculture, University of Massachusetts, Amherst, Massachusetts 01003, United States; 4 Key Laboratory of Solid Waste Treatment and Resource Recycle, Ministry of Education, Southwest University of Science and Technology, Mianyang 621010, China

## Abstract

The uptake of organic chemicals by plants is considered of great significance as it impacts their environmental transport and fate and threatens crop growth and food safety. Herein, the dependence of the uptake, penetration, and distribution of sixteen polycyclic aromatic hydrocarbons (PAHs) on the morphology and micro-structures of cuticular waxes on leaf surfaces was investigated. Plant surface morphologies and wax micro-structures were examined by scanning emission microscopy, and hydrophobicities of plant surfaces were monitored through contact angle measurements. PAHs in the cuticles and inner tissues were distinguished by sequential extraction, and the cuticle was verified to be the dominant reservoir for the accumulation of lipophilic pollutants. The interspecies differences in PAH concentrations cannot be explained by normalizing them to the plant lipid content. PAHs in the inner tissues became concentrated with the increase of tissue lipid content, while a generally negative correlation between the PAH concentration in cuticles and the epicuticular wax content was found. PAHs on the adaxial and abaxial sides of a leaf were differentiated for the first time, and the divergence between these two sides can be ascribed to the variations in surface morphologies. The role of leaf lipids was redefined and differentiated.

Polycyclic aromatic hydrocarbons (PAHs) are widely distributed and persistent organic contaminants in the environment^1^,^2^,^3^,^4^,^5^. Of the total PAH emission into the urban environment, 40% is scavenged by vegetation via dry and wet deposition^6^,^7^. The dynamic exchange of organic chemicals occurs frequently at the plant surfaces and under the impacts of both complex environmental conditions and biological properties of the plant^8^,^9^,^10^,^11^. Plant uptake of organic chemicals is considered of great significance as it impacts the environmental transport and fate of PAHs and threatens crop growth^3^,^12^,^13^,^14^. Due to the large surface area, the foliar interface is considered as the main access for organic chemical accumulation^3^. The uptake of organic chemicals at plant surfaces have been widely investigated with respect to their diffusion and penetration, and plant lipids have been identified as the key chemical component for the assimilation of organic pollutants^12^,^13^,^15^,^16^,^17^,^18^,^19^,^20^,^21^. However, the organic contamination levels of plants in the same/similar local environment vary considerably, and this interspecific difference cannot be solely explained by the content of leaf lipids^12^,^22^,^23^,^24^.

Organic chemicals have been found to distribute unevenly in foliage with the majority of them being accumulated in the outermost polyester skin of the leaf, i.e., cuticle^14^,^16^,^20^,^25^,^26^. Cuticle is recognized as the very first barrier protecting plants from external impacts, and both the outer structures and inner chemical compositions are critical to this process^27^,^28^,^29^,^30^,^31^. Towards the outermost lipid membrane, the cuticular lipids can be differentiated as extractable lipids (waxes) and un-extractable lipids (polymeric lipids) via sequential separation with organic solvent extraction and depolymerization, and polymeric lipids have been identified as the most effective sink, trapping organic pollutants from entering the inner parts of the plant^11^,^16^,^25^,^32^. However, measurements through batch sorption experiments have inevitably neglected the elaborate structure of plant surfaces, and up to now, the interspecies difference in foliar uptake of organic chemicals still remains unresolved^33^,^34^.

Foliar surfaces, specifically cuticular matrices, are assembled with vertical heterogeneity in the chemical compositions and lateral diversity in the epicuticular wax morphologies^11^,^25^,^27^,^30^,^35^,^36^. Numerous studies have focused on these kinds of multifunctional surfaces and thus biomimic materials development has been inspired^37^,^38^,^39^. Leaves with a larger surface area are more advantageous in organic pollutant accumulation that those with a smaller area^3^. Furthermore, it has been reported that leaves exhibiting pubescence (hairiness) had significantly higher ΣPAH concentrations than hairless leaves^40^. Since PAHs are hydrophobic compounds, airborne PAHs are deposited on foliar surfaces mainly by dry deposition (gaseous and particulate-bound forms)^3^. Surface stereo micro-structure and hydrophobicity could have effects on the foliar uptake of organic pollutants. However, there has been little research on this aspect.

The objective of this study is to explore the roles of morphologies of foliar surfaces in the uptake and penetration of organic pollutants. To achieve this, plant leaves of six shrub species were sampled. The surface morphologies and properties were examined through scanning electron microscopy (SEM) and contacting angle (CA) measurements. A sequential separation method was applied to extract PAHs in the leaf cuticles and inner tissues, and PAHs on the adaxial and abaxial leaf surfaces were differentiated. Finally, the driving force of organic pollutant diffusion from the cuticles to the inner tissues was addressed.

## Results and Discussion

### Plant surface morphologies and characterizations

The water, epicuticular wax and extractable lipid contents of six selected plant leaves are presented in Table 1, and significant interspecies differences were found. For example, *Hypericum monogynum* foliage was the moistest with a water content of 60.36%; meanwhile, it also contained the highest content of extractable lipids (4.31%). Due to the relatively low content of epicuticular wax (0.88%), its inner tissue lipid content (3.43%) was the highest among the selected leaf species. By contrast, *Pinus massoniana* contained the lowest content of water (33.72%); *Mahonia fortunei* foliage contained the lowest contents of both epicuticular waxes (0.30%) and extractable lipids (0.79%). Due to the lack of lipids, *Mahonia* probably had the most “clean” foliage in terms of organic pollutants in the sampling site, while *Hypericum* foliage would be an ideal candidate as a passive sampler for organic pollutants and bio-indicator for the atmospheric pollution level of the sampling site. Additionally, it was concluded that water-soluble contaminants were mainly reserved in the plant-water phase^12^,^21^,^41^. Herein, compared to *Hypericum* foliage which excelled in both water and lipid contents, *Pinus* would be an ideal candidate as a selective passive sampler mainly accumulating organic pollutants because its needles had the lowest water content (33.72%) and a relatively high lipid content (3.06%).

**Figure 1:**
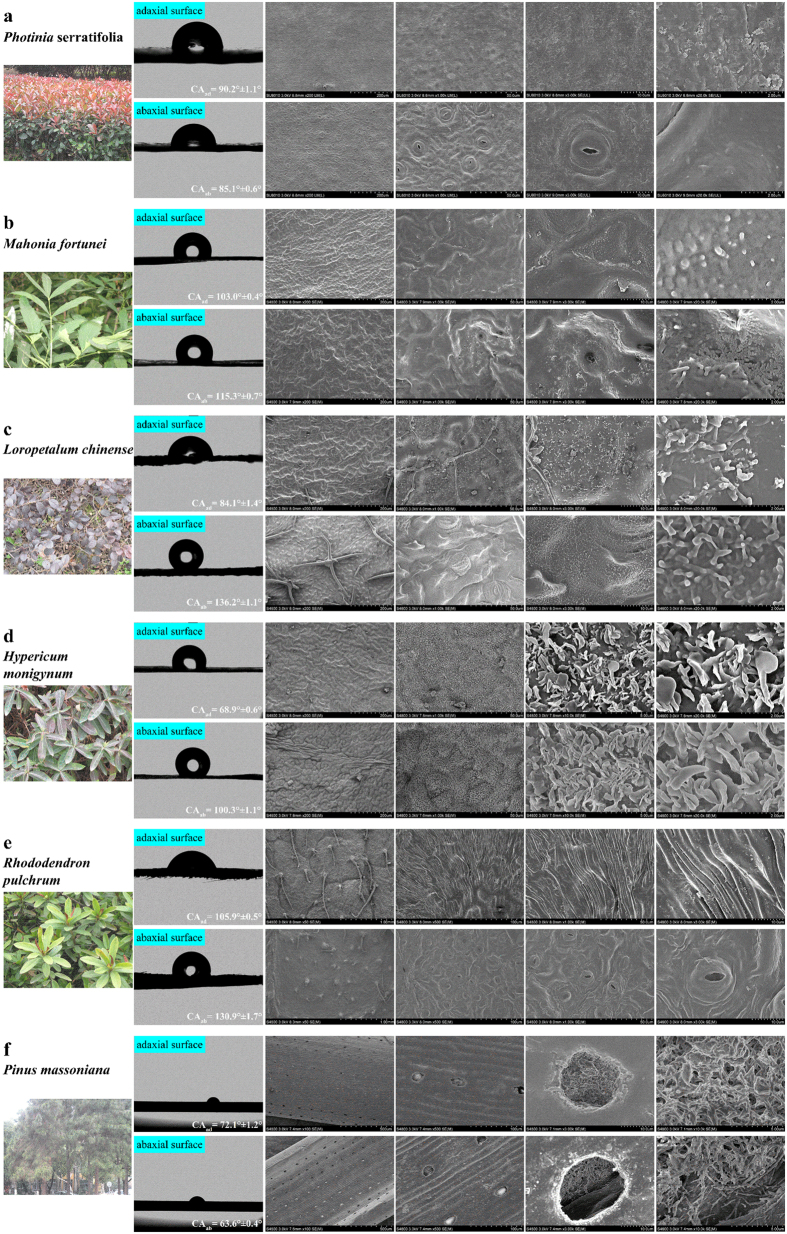
Photographs, surface morphologies from SEM and contact angle (CA) measurements of selected foliar species: *Photinia serratifolia* (**a**), *Mahonia fortunei* (**b**)*, Loropetalum chinense* (**c**), *Hypericum monogynum* (**d**), *Rhododendron pulchrum* (**e**) and *Pinus massoniana* (**f**). Upper rows represent adaxial surfaces and lower rows represent abaxial surfaces. SEM photographs are presented in different magnifications (left to right: 200×, 1000×, 3000×, and 10000×).

The surface morphologies on both the adaxial and abaxial sides of selected plant leaves were observed under SEM, and the CAs were recorded (Fig. 1). Abundant 2D and 3D wax micro-structures and significant interspecies differences were observed, and obvious variations between the adaxial and abaxial surfaces were also demonstrated. Generally, abaxial surfaces were rougher than adaxial ones. Except for *Pinus* needles, which had stomas on both sides, the other five leaf species were all hypostomatic. CA is used to describe the extent to which a surface can become wet^30^. Among the selected leaf species, the most hydrophobic surface belonged to the abaxial side of *Loropetalum* (average CA of 136.2°) and *Rhododendron* (average CA of 130.9°). Both of these two leaf species have hairs attached to their abaxial surfaces. According to the literature, these two species could accumulate more organic pollutants than hairless ones. The most hydrophilic surface was the adaxial side of *Hypericum* (average CA of 68.9°), which was probably related to its high water content (60.36%).

Specifically, as presented in Fig. 1a, the adaxial surface of *Photinia* was slightly wrinkled with scattered epidermis wax papillae, and rough wax micro-structures were found on this side. The abaxial side undulated and was relatively smooth with stomas spread on the surface, while no obvious wax stereoscopic structure was observed. The hydrophobicity of both sides showed very limited difference with the CA varying less than 5°. The leaf surfaces of both sides of *Mahonia* undulated and very rigid, irregular wax papillae were observed in the shallow pits on both sides (Fig. 1b). Stomas were distributed right in those shallow pits on the abaxial surfaces, and the wax papillae structures were denser than those on the adaxial surfaces. The CA of the abaxial surface (115.3° ± 0.7°) was slightly larger than that of the adaxial surface (103.0° ± 0.4°), indicating higher hydrophobicity on the abaxial surface.

For *Loropetalum* (Fig. 1c), both the adaxial and abaxial sides of this foliage were covered by dense rodlet-like wax, and sparse cross-shaped hairs were attached to the surfaces. Moreover, the micro-structures of cuticular waxes on the abaxial side were more regular than those on the adaxial side. The very different CAs of the adaxial and abaxial sides (84.1° ± 1.4° and 136.2° ± 1.1°, respectively) suggested a significant difference of hydrophilicity/hydrophobicity on the opposite sides. Impressively, dense wax micro-plates were found to be attached to the surfaces on both sides of *Hypericum* (Fig. 1d). These wax crystals varied greatly in size and shape and have irregular shapes with wavy outside edges. They were closely interconnected and completely covered the undulated surfaces. On the abaxial surface, stomas were hidden under the dense wax plates, which can be recognized under careful observation. Although direct observation illustrated only a slight difference between the opposite sides, CA measurements revealed that the abaxial surface (100.3° ± 1.1°) was much more hydrophobic than the adaxial surface (68.9° ± 0.6°).

On both sides of *Rhododendron*, very deep folds were oriented towards the hairs attached to the epidermis, and granulated wax structures were found in the fissures of those folds (Fig. 1e). The CA of the abaxial surface (130.9° ± 1.7°) was larger than that of the adaxial surface (105.9° ± 0.5°). For the needles of *Pinus* (Fig. 1f), the abaxial surface was rougher than the adaxial surface, while the CA of the abaxial surface (62.6° ± 0.4°) was significantly smaller than that of the adaxial surface (72.1° ± 1.2°). A number of oval or round-shaped micro-chambers were found rowed in the longitudinal channels on both sides of the needles. In these epistomatal chambers, rod-like epicuticular wax structures were coalesced with each other, forming complex wax network structures, while no apparent florin ring was observed on the pine needles^42^. As the needles of *Pinus* were less than 2 mm wide, the contact diameter of 3 μL of distilled water was too large; therefore, the CAs of *Pinus* were measured using 0.6 μL of distilled water. It was reported that the CAs started to decrease with droplet volumes of approximately 2 and 3.7 μL on medium- and low-adhesive surfaces^43^. As a consequence, CAs measured for *Pinus* can only be used as a reference to investigate the hydrophobic difference between its adaxial and abaxial surfaces, but they cannot be applied for comparison among the leaf species.

### PAH distribution characteristics in leaves

**Figure 2:**
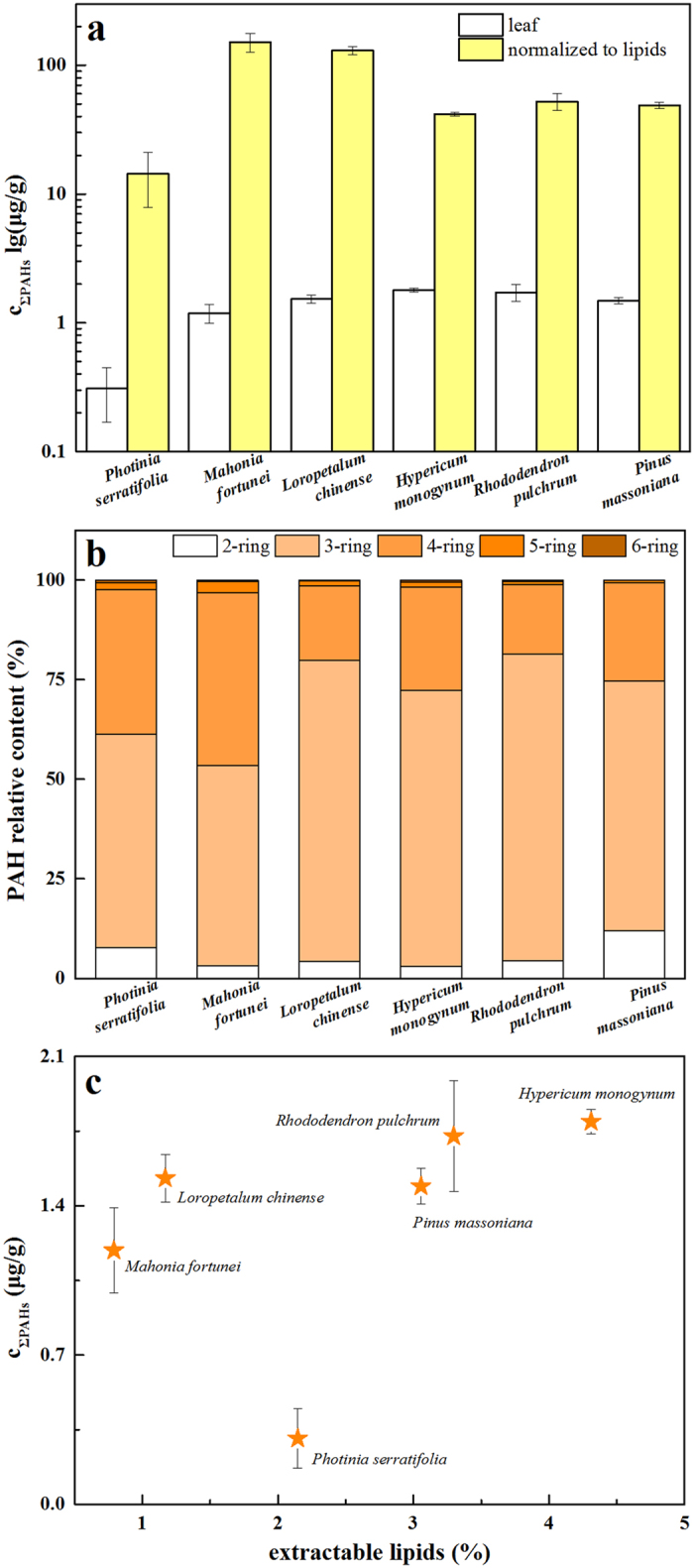
ΣPAH concentrations in the leaves and extractable lipids of selected plant species (**a**), PAH relative contents sorted by rings in the leaves (**b**), and correlation between ΣPAH concentrations and the contents of extractable lipids (**c**).

**Figure 3:**
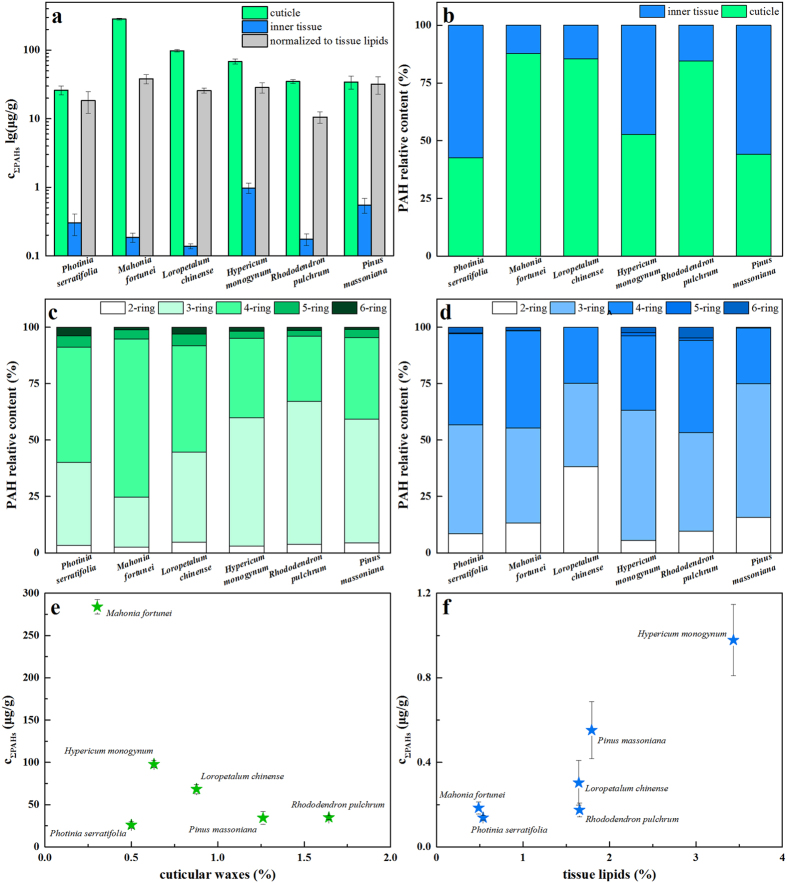
ΣPAH concentrations in leaf cuticles, inner tissues and tissue lipids (**a**), ΣPAH relative contents sorted by rings in cuticles (**b**) and inner tissues (**c**), ΣPAH relative contents in cuticles and inner tissue (**d**), and correlations between ΣPAH concentrations (**e)** and lipid contents (**f**).

The concentrations and distributions of ΣPAHs in selected leaf species are presented in Fig. 2. Even though the sampling site was very small in area, which suggested a similar air pollution level, ΣPAHs uptake by the plant leaves showed significant differences among the species (Fig. 2a). Among the selected leaf species, *Hypericum* with the densest wax structure and highest lipid content accumulated the most PAHs in terms of concentration (1.80 μg/g). However, from our hypothesis mentioned above, the most “clean” species was *Photinia* (0.31 μg/g), rather than *Mahonia* (1.19 μg/g) with the least lipid content, which, in contrast, even excelled at accumulating pollutants in terms of the normalized-to-lipid concentration (151 μg/g). The concentrations of 16 separated PAHs in the selected plant species are presented in Fig. S-1. Each plant species showed a different ability for the uptake of PAHs. The measured concentrations of PAHs were similar to those reviewed in the literature (1~4000 ng/g dry weight)^3^. The distributions of PAHs sorted by rings are presented in Fig. 2b, and 3,4-ring PAHs composed the highest uptake amount of ΣPAHs. Gaseous 2-ring PAHs usually undergo frequent exchanges at plant surfaces, so it had a very small fraction of ΣPAHs. Meanwhile, the mobility of organic substances within the plant cuticle was believed to be size dependent, so an increase in the molecular weight resulted in a decrease in mobility^44^. Hence, heavy PAHs with 5 and 6 rings had more difficulty entering the plant organism and as a consequence had negligible fractions. The correlation between ΣPAH concentrations and lipid content is demonstrated in Fig. 2c. Except for that of *Photinia*, there was a slight elevation in the ΣPAHs accumulation capacities of selected leaves with the increase of lipid content. Although it contained a relatively medium content of extractable lipids (2.15%), ΣPAH deposition was unfavorable on the leaf surface of *Photinia*. This result suggested that only considering the content of foliar lipids was not sufficient to explain the uptake and distribution characteristics of atmospheric PAHs into these leaves.

PAHs uptake by leaves were further differentiated into those in the cuticles and inner tissues. Interspecific differences between the ΣPAH distribution in the cuticles and inner tissues were demonstrated in the concentrations (Fig. 3a) and relative contents (Fig. 3b,c,d). The cuticles showed extraordinary capabilities for trapping PAHs, and the diffusion of organic pollutants into the inner part of the leaves was limited (Fig. 3a). The relative contents of PAHs in the cuticles and inner tissues varied, among which *Mahonia, Loropetalum*, and *Rhododendron* demonstrated an overwhelming preference for PAH accumulation in their cuticles while the other three species had approximately equivalent or even less capability of PAH accumulation in the inner tissues (Fig. 3d). In cuticles, 3- and 4-ring PAHs were still the most abundant components (>90%), while the circumstances changed in the cases of inner tissues, where 2-ring PAHs showed an elevated amount. Obviously, this was due to the increased difficulties of PAHs with more rings to diffuse into the inner tissues, rather than the increased penetration of 2-ring PAHs with low molecular weights, which was verified in Fig. S-2. Each individual PAH was assigned to the differentiation of distribution and every PAH in the cuticle exceeded that in the inner tissue.

The correlations between PAH uptake and differentiated lipids (regarding cuticular wax and inner tissue lipids) were explored (Fig. 3e,f). Interestingly, accumulation of PAHs in cuticles decreased with increasing cuticular wax content. For example, the leaf with the lowest content of cuticular wax (0.30%), i.e., *Mahonia*, accumulated ΣPAHs with the highest concentration in the cuticle (284 μg/g). The exception to this correlation is *Photinia*, which contained the second lowest content of cuticular wax (0.50%) but accumulated ΣPAHs with the lowest concentration in the cuticle (26.02 μg/g), which was an order of magnitude lower than that of *Mahonia*. In contrast to that found for cuticular wax, a general positive correlation between ΣPAH concentration in the inner tissue and tissue lipid content was found. In the case of *Mahonia*, the lowest content of tissue lipid (0.49%) led to the second lowest accumulation of ΣPAHs in its inner tissue (0.19 μg/g). *Hypericum* with the highest tissue lipid content (3.43%) had the most concentrated PAHs in the inner tissue (0.98 μg/g).

To the best of our knowledge, the differences between epicuticular wax and inner tissue lipids in the uptake of organic pollutant were two fold. First, the accessibility of organic pollutants to these two components was successive. The organic pollutants deposited on the epicuticular wax first and then diffused into the inner tissue. The organic pollutants in the inner tissues came from the transportation of those in the epicuticular wax. Nonetheless, this process only occurred during creation of the pollutant concentration gradient, which should not have an influence on the affinity of lipid to organic pollutants. Second, the epicuticular wax encountered by organic pollutants primarily had abundant stereo-structures, while the tissue lipid in the inner part of the plant was mainly in amorphous form. As mentioned before, unlike the other five species, the smooth surface morphology of *Photinia* was not advantageous for the uptake of PAHs in gaseous and liquid forms due to its relatively small surface area per unit mass. Furthermore, it may allow the particulates bundled with PAHs to be easily washed off by rainfall, while those with impressive stereo wax structures can contribute to the trapping of these particulates on their surfaces, and diffusion of PAHs on the particulates to the inner parts may therefore be facilitated. Plants evolve to adapt to the environment. The high efficiency of *Mahonia* as a pollutant barrier can be ascribed to its compact and least lipophilic surfaces (0.30%). PAHs cannot easily penetrate through this resistant armor, and they remain trapped in the cuticular stereo matrix on the surface. The source of concentrated PAHs in the tissue lipid of *Hypericum* can therefore be attributed to the accessible epicuticular wax structures that continuously trap deposited PAHs and transport them into the inner organic pollutant reservoir. For *Pinus* with epistomatal chambers that can be opened up, the second most concentrated PAH accumulation in its inner tissue (0.55 μg/g) would be expected.

**Figure 4:**
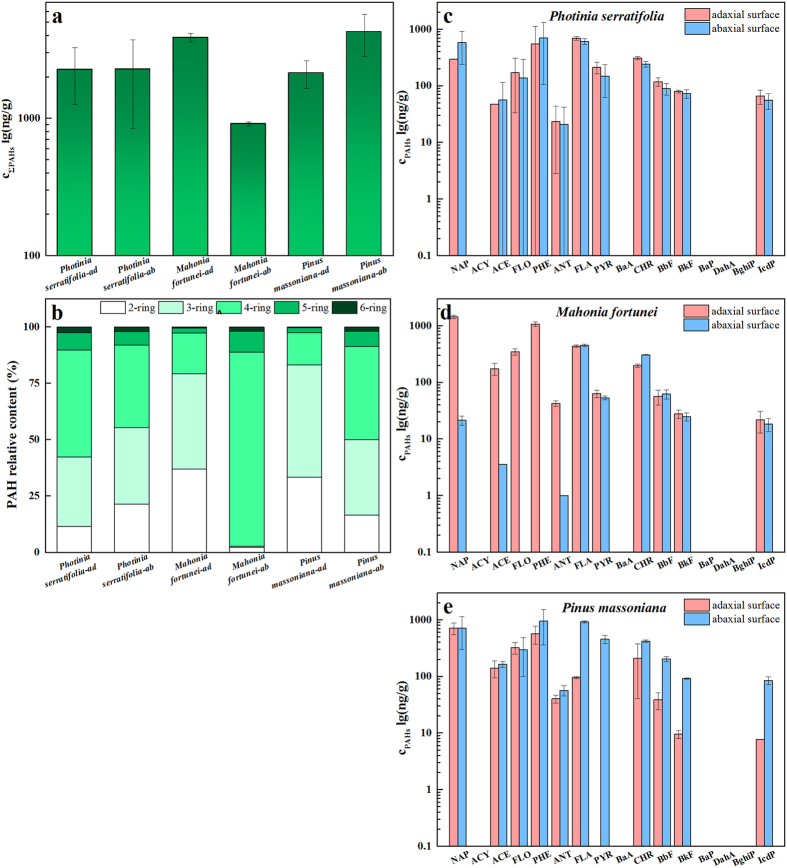
ΣPAH concentrations on adaxial and abaxial surfaces of selected plant species (**a**), PAH relative contents sorted by rings on the adaxial and abaxial surfaces (**b**), and concentrations of individual PAHs in the adaxial and abaxial surfaces of leaf species of *Photinia serratifolia* (**c**), *Mahonia fortunei* (**d**) and *Pinus massoniana* (**e**).

Abundant epicuticular wax micro-structures on plant surfaces, accompanied by the cuticular matrix beneath, have important functions in protecting the plant from outer harm and pessimal stimulations. The PAH distributions on adaxial and abaxial leaf surfaces were initially examined and are presented in Fig. 4. For *Photinia*, PAH contamination on the adaxial surfaces (2.26 μg/g) was equivalent to that on the abaxial surfaces (2.28 μg/g), which was in good agreement with the similar surface morphologies and hydrophobicities of both sides.

A slight difference between both sides is shown in Fig. 4b,c with an elevated proportion of 2-ring PAHs on the abaxial surfaces. For *Mahonia*, PAHs that accumulated on the adaxial surface (3.87 μg/g) were almost four times higher than those on the abaxial surface (0.91 μg/g). A significant difference in the distribution was demonstrated (Fig. 4b,d), and PAHs with 2 and 3 rings were mostly accumulated on the adaxial side, while those with more than three rings deposited on the abaxial side. However, SEM observations showed little advantage of the adaxial waxes trapping the light and intermediate PAHs. It is noteworthy that the adaxial surface of *Mahonia* was dark green compared to the abaxial surface and this may lead to the divergence in photochemical reactions. However, currently, the source of this variation cannot be determined for sure. In contrast to *Mahonia*, as presented in Fig. 4b,e, only half of the PAHs on the abaxial surfaces (3.73 μg/g) of *Pinus* accumulated on the adaxial surfaces (2.14 μg/g) in terms of concentration, and the distributions also differed. PAHs with medium and high molecular weights dominated the accumulation on the rough adaxial surface, which was considered to be favorable for PAH capture.

### Driving force for PAH penetration through cuticles

**Figure 5:**
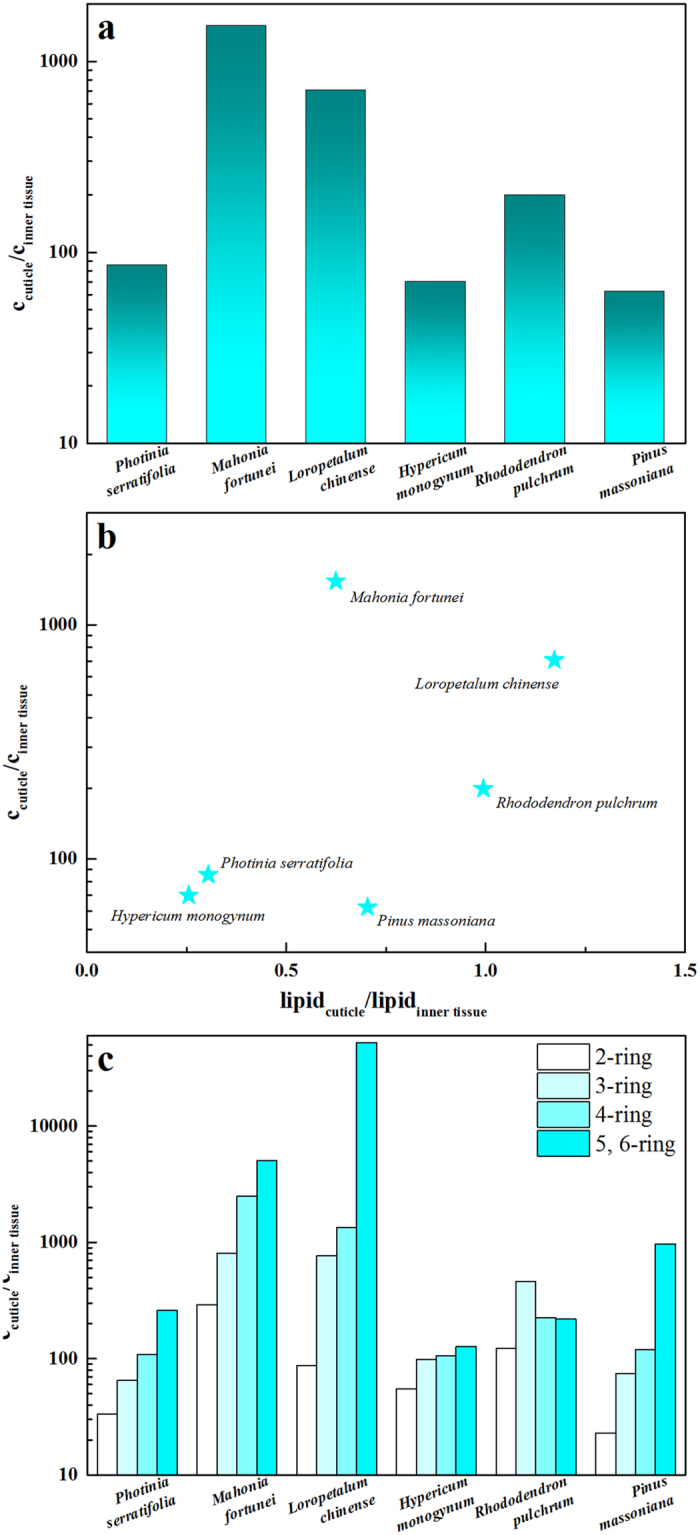
Concentrations ratios of ΣPAHs in cuticles to those in inner tissues (**a**), correlation between ΣPAH concentration ratios and lipid gradients (**b**), and concentration ratios of 2-, 3-, 4-, 5-, and 6-ring PAHs in cuticles to those in the inner tissues (**c**).

Up to now, lipid as the main reservoir for organic pollutants accumulation was verified and the contributions of lipid micro-structures were addressed as an important factor for regulating the uptake behavior of organic pollutants in addition to the lipid content. For a deeper understanding of the organic pollutant uptake process and diffusion behavior, the diffusion driving force is introduced. It was widely recognized that the fugacity gradient generated between the plant surface and the inner tissue was the driving force for organic pollutant diffusion^11^,^25^. As presented in Fig. 5a, the concentration gradients varied widely among the selected plant species and were in good agreement with their surface morphologies. To be specific, for the *Mahonia* leaf, which is covered by a compact and rigid cuticle armor, the sharpest concentration gradient (with a ratio of 1535) was created, which consequently provided the highest diffusion driving force. In contrast, for *Hypericum* and *Pinus,* which contained trapping epicuticular structures and opened-up epistomatal chambers effective for transporting PAHs into the inner plant parts, the driving force for diffusion was the lowest (concentration gradient with ratios of 69.9 and 62.2, respectively).

The correlation between PAH gradients and lipid gradients is shown in Fig. 5b. If only the lipid content matters, a general positive correlation would be observed between PAH gradients and lipid gradients. However, for *Hypericum* and *Pinus*, which share distinct lipid gradients, similar pollutant gradients were produced. Additionally, *Mahonia* had the highest PAH gradient, although it did not possess any overwhelming lipid gradient. These results provided further evidence on the insufficiency of characterizing the penetration behavior solely by the lipid content. In addition to the characteristics of the transport medium regulating the diffusion behavior, the properties of transported compounds could also impact the uptake process. The size selectivity was clearly demonstrated in Fig. 5c, and the uptake of PAH with different rings showed varied partitions between the surface and inner tissue, which was reflected in the different concentration gradients.

**Figure 6:**
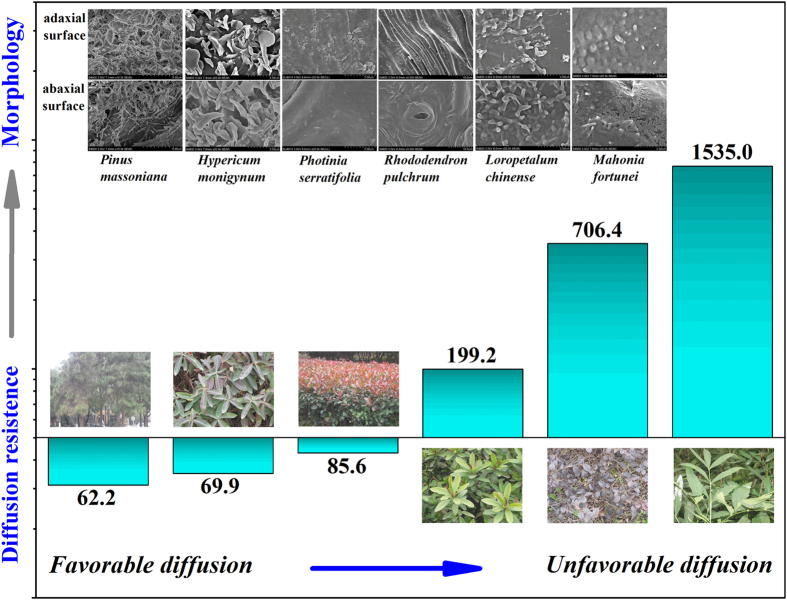
Plant uptake and diffusion resistance of organic pollutants were dominated by the leaf surface morphologies and micro-structures of epicuticular wax rather than just their lipid contents.

In summary, plant uptake of PAHs was a complex process. Wide differences were discovered among the selected plant species. *Hypericum* and *Pinus* were considered to be the superior reservoirs for organic pollutant accumulation and can be further applied as effective passive samplers for organic pollution monitoring. *Mahonia* showed an extraordinary capability in blocking organic pollutants from penetrating into the inner tissue of the leaf. The plant surface morphology, specifically the cuticular wax micro-structure, was found to be an essential factor regulating the deposition, distribution, and penetration of organic pollutants in and across plant cuticles (Fig. 6). This study provided a theoretical reference to precisely predict the plant uptake of HOCs and to design a smart biomimic material for HOC passive sampler.

## Materials and Methods

### Plant materials and characterization

Mature leaves of six shrub plants (*Photinia, Mahonia, Loropetalum, Hypericum, Rhododendron* and *Pinus*) were sampled in a field area within 50 meters in diameter from Xixi campus, Zhejiang University, China. The leaves were collected and washed with distilled water to remove dust from the surfaces. The air-dried leaves were weighed, and the water, wax, and extractable lipid contents were further characterized. The detailed procedure is presented in the Supporting Information. Foliar surface morphologies of the adaxial and abaxial sides were observed with a field emission scanning electron microscope (FE-SEM, SK4800 & SU8010; Hitachi, Tokyo, Japan). Specimens for SEM observation were prepared following a modified method reported earlier^25^. Contact angles (CA) were determined by the sessile-drop method with distilled water (3 μL) on a video-based contact angle measuring device (OCA20, Filderstadt, Germany) at room temperature. Average CA values were obtained by measuring two different positions in one sample, and leaf veins were avoided. Because the plant samples were collected from a field area rather than from an exposure experiment in the lab or greenhouse, the plants were exposed to PAHs in the atmospheric environment. However, the PAH concentrations in the atmosphere were not monitored, and their existing forms were not differentiated (vapor phase or sorbed by dusts in air), since the specific objective of the study is to elucidate the dependence of plant uptake and diffusion of PAHs on the leaf surface morphology and micro-structures of cuticular waxes. All of the leaves used in the current study were collected once, and immature leaves were avoided. According to the horticulture interval time, these leaves were contaminated for approximately 6 months during their growth.

### Extraction of PAHs in cuticles and inner tissues of plant leaves

PAHs in the leaves were obtained directly through organic solvent extraction. The leaf samples were cut into pieces and freeze-dried. The dehydrated samples were then weighed, and PAHs were extracted with a 1:1 mixture of n-hexane and acetone under a circulating water bath in an ultrasonic cleaning instrument for 30 min. The procedure was repeated 3 times by collecting the extract and adding new solvent each time. The gathered extracts were added to 30 μL dimethyl sulfoxide (DMSO) to protect the PAHs from evaporation and were then concentrated with a vacuum rotary evaporator at 40 °C. The concentrates were transferred with n-hexane to a silica (2.5 g) filled column and eluted with 25 mL of 1:1 mixture of n-hexane and dichloromethane for purification. The eluate with 30 μL DMSO added was concentrated to near dryness by vacuum rotary evaporation. Then, it was transferred with 3 mL of acetonitrile and filtered through a syringe filter (0.22 μm) to a sample bottle for high-performance liquid chromatography (HPLC) analysis.

PAHs in the cuticles and inner tissues were extracted by applying a modified sequential extraction procedure^45^. Specifically, air-dried intact leaves (~6 g) were extracted with dichloromethane (150 mL) in a glass flask for 2 min with gentle shaking. Out of the 150 mL extract, 50 mL was used for the measurement of cuticular wax contents. The other 100 mL was concentrated through the same procedure as the extraction and determination of leaf PAHs. The leaf samples after the extraction of cuticular PAHs were cut into pieces and freeze-dried for the successive extraction of PAHs in the inner tissues. The procedures for the concentration of PAH extraction in the inner tissues were the same as those for the leaves.

The PAHs in the adaxial and abaxial cuticles were extracted by the following method. Briefly, cuticles on the adaxial and abaxial leaf surfaces were obtained by scraping or peeling from the leaf surfaces using a sharp blade. This method was attempted on all six leaf species, and it was practical on only three of them: *Photinia, Mahonia*, and *Pinus*. The obtained biomaterials were weighed and freeze-dried. The successive procedures for PAH extraction were the same as those mentioned above. Quality control was performed to verify the feasibility of the sequential extraction method. The average recovery rates of the other 15 PAHs were within 83.0–119.2%, except for naphthalene in which the average recovery rate was merely half (44.67%). Therefore, the concentration of naphthalene was underestimated.

### Determination of PAH concentrations

Samples of cuticular waxes and inner tissues, as well as the biomaterials obtained from the adaxial and abaxial leaf surfaces, were subject to PAH measurement on an HPLC. The 16 PAHs include naphthalene, acenaphthene (ACE), acenaphthylene, fluorene (FLO), phenanthrene (PHE), anthracene (ANT), fluoranthene (FLA), pyrene (PYR), benz(a)anthracene (BaA), chrysene (CHR), benzo(b)fluoranthene (BbF), benzo(k)fluoranthene (BkF), benzo(a)pyrene (BaP), dibenz(a, h)anthracene (DahA), benzo(g, h, i)perylene (BghiP), and indeno(l,2,3-cd)pyrene (IcdP). PAHs were detected by a fluorescence detector (FLD). The column temperature was set at 30 °C, and the mobile phase was a mixture of acetonitrile and distilled water with periodically varied ratios. The gradient elution program is presented in Table S1. The flow rate was set to 1 mL/min, and the injection volume of each sample was 15 μL. The excitation wavelengths of the 16 PAHs are presented in Table S2. The detection limit of individual PAH ranged from 0.217 to 2.002 μg/kg.

## Additional Information

**How to cite this article**: Li, Q. *et al*. Dependence of Plant Uptake and Diffusion of Polycyclic Aromatic Hydrocarbons on the Leaf Surface Morphology and Micro-structures of Cuticular Waxes. *Sci. Rep.* **7**, 46235; doi: 10.1038/srep46235 (2017).

**Publisher's note:** Springer Nature remains neutral with regard to jurisdictional claims in published maps and institutional affiliations.

## Supplementary Material

Supporting Information

## Figures and Tables

**Table 1:** Primary componential contents of six selected plant species.

plant species	water%^a^	epicuticular waxes%^b^	extractable lipids%^b^
*Photinia serratifolia*	57.55	0.50	2.15
*Mahonia fortunei*	51.35	0.30	0.79
*Loropetalum chinense*	44.33	0.63	1.17
*Hypericum monogynum*	60.36	0.88	4.31
*Rhododendron pulchrum*	55.61	1.64	3.30
*Pinus massoniana*	33.72	1.26	3.06

^a^Water contents were calculated upon leaf fresh weight basis.

^b^Wax and extractable lipid contents were calculated upon leaf dry weight basis.
