# Supplementary material for: Dependence of Plant Uptake and Diffusion of Polycyclic Aromatic Hydrocarbons on the Leaf Surface Morphology and Micro-structures of Cuticular Waxes
Source: Sci Rep. 2017 Apr 10;7:46235. doi: 10.1038/srep46235 (PMC5385540; doi:10.1038/srep46235)
Supplement: Supporting Information [file srep46235-s1.pdf]

Supporting Information Section for *Scientific Reports*

Dependence of Plant Uptake and Diffusion of Polycyclic Aromatic Hydrocarbons on the Leaf Surface Morphology and Micro-structures of Cuticular Waxes

Qingqing Li[†, ‡], Yungui Li[§], Lizhong Zhu[†, ‡], Baoshan Xing[#], and Baoliang Chen[†, ‡,*]

† Department of Environmental Science, Zhejiang University, Hangzhou 310058, China

‡ Zhejiang Provincial Key Laboratory of Organic Pollution Process and Control, Hangzhou 310058, China

§ Key Laboratory of Solid Waste Treatment and Resource Recycle, Ministry of Education, Southwest University of Science and Technology, Mianyang 621010, China

Stockbridge School of Agriculture, University of Massachusetts, Amherst, Massachusetts 01003, United States

* Corresponding Author E-mail: blchen@zju.edu.cn

Phone: 0086-571-88982587

Fax: 0086-571-88982587

Supporting Information consists of 8 pages including this one.

There are two Tables and two Figures.

November 12, 2016

Measurements for the water, wax, and extractable lipid contents.

Air-dried leaves were weighed and oven-dried for 24 h at 105 °C to remove water. The completely dried leaves were weighed and the water content was calculated based on mass balance. Air-dried leaves were weighed and directly placed into 50 mL of dichloromethane. After 2 minutes of stirring, the solvent was filtered (Sartorius AG 0.45 μm) and transferred to pre-weighed vials. Cuticular wax was obtained after solvent evaporation, and the wax content was calculated based on mass balance. Oven-dried leaves were weighed and placed into vials containing a mixture of n-hexane and acetone (v/v, 1/1). The vials were then sealed and placed in an ultrasound water bath for lipid extraction. Extractable cuticular lipids were measured through ultrasonic extraction. Approximately 6 g of air-dried (25 °C, 1 h) leaf samples were cut into pieces and freeze-dried under vacuum. The dehydrated samples were extracted under a circulating water bath in an ultrasonic cleaning instrument using 1:1 mixture of n-hexane and acetone for 1 h, and the procedure was repeated 3 times by collecting the extract and adding new solvent each time. The extract was weighed after the solvent evaporated completely. For each plant species, three replicate measurements of the water, wax and extractable lipid contents were conducted.

Preparation procedure of specimens for SEM observation

The specimens for SEM examination were prepared according to a modified method [1]. In detail, clean and fresh leaves were placed in liquid nitrogen (-196 °C) for instant preservation of morphology and then desiccated under vacuum. Except for pine needles, the squares of dry leaves (approximately 5 mm × 5 mm) were excised, avoiding the mid rib areas to give a relatively flat surface. The pine needles were only cut into segments of approximately 5 mm long. Both the adaxial and abaxial surfaces were mounted on a metal holder using two-sided adhesive carbon tape and platinum-coated twice under an argon

atmosphere in a sputter-coater (E-1030; Hitachi, Tokyo, Japan), each time for 30 seconds. The inclination angle of the stub was adjusted to make sure the platinum layer coated the microstructures of the cuticular waxes completely. The specimens were then examined with a field emission scanning electron microscope (FE-SEM, S-4800 & SU8010; Hitachi, Tokyo, Japan) operated at an accelerating voltage of 3 kV. The nomenclature of the wax morphology proposed by Barthlott et al. was adopted in this study.[2]

Table S1

Gradient elution program for the analysis of the 16 PAHs.

Time (min)	0.00	3.00	20.00	30.00	40.00	53.00	55.00
Acetonitrile (%)	40.0	40.0	60.0	60.0	10.0	10.0	40.0

Table S2

Excitation and emission wavelengths of PAHs.

PAH	λ_{ex} (nm)	λ_{em} (nm)
naphthalene (NAP)	220	325
acenaphthene (ACE)	220	325
acenaphthylene (ACY)	220	315
fluorene (FLO)	220	315
phenanthrene (PHE)	244	360
anthracene (ANT)	244	400
fluoranthene (FLA)	237	460
pyrene (PYR)	237	385
benz(a)anthracene (BaA)	277	376
chrysene (CHR)	277	373
benzo(b)fluoranthene (BbF)	255	420
benzo(k)fluoranthene (BkF)	255	420
benzo(a)pyrene (BaP)	255	420
dibenz(a,h)anthracene (DahA)	300	415
benzo(g,h,i)perylene (BghiP)	300	415
indeno(l,2,3-cd)pyrene (IcdP)	250	495

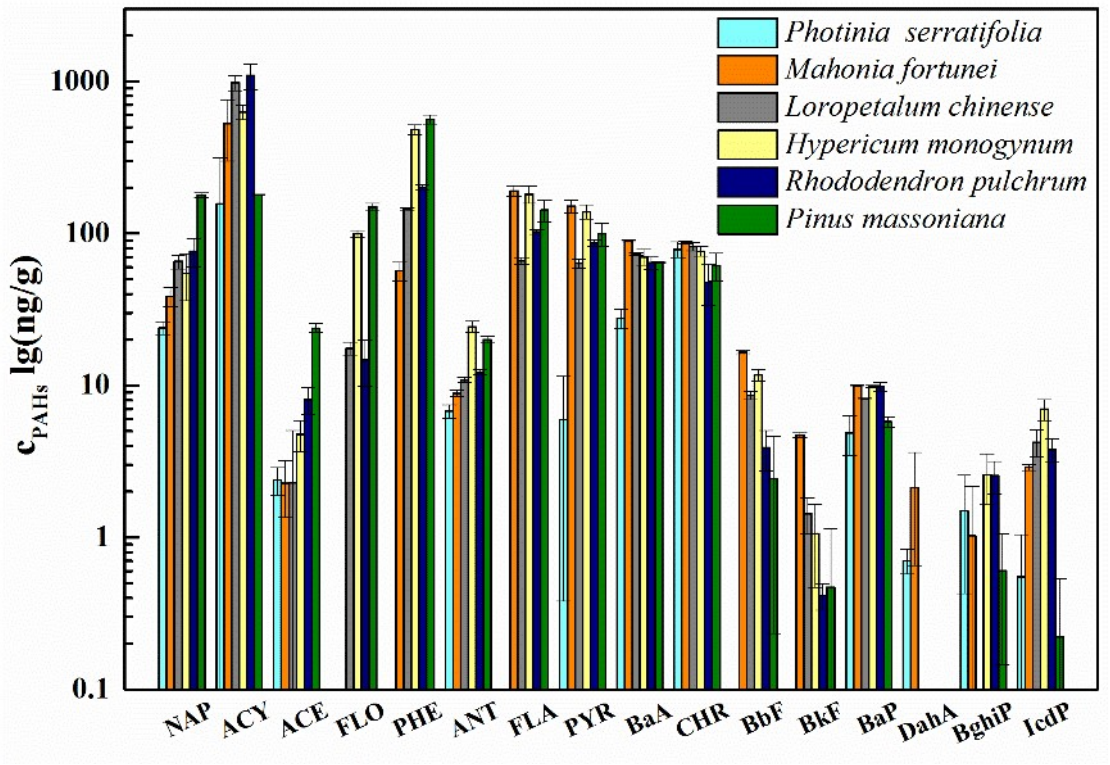

Figure S1. Concentrations of individual PAHs in the leaves of selected plant species.

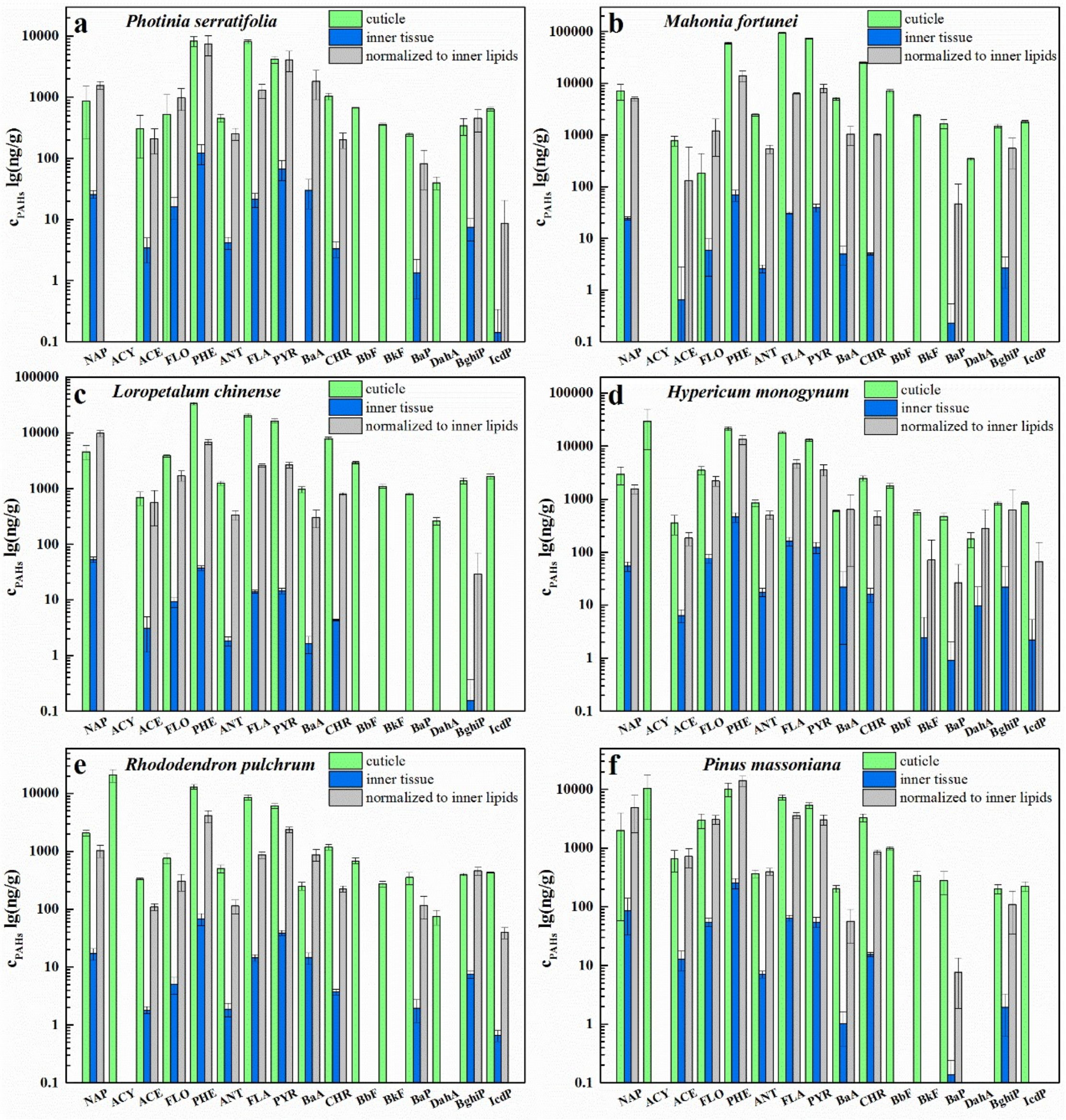

Figure S2. Concentrations of individual PAHs in the cuticles and inner tissues of *Photinia serratifolia* (a), *Mahonia fortunei* (b), *Loropetalum chinense* (c), *Hypericum monogynum* (d), *Rhododendron pulchrum* (e), and *Pinus massoniana* (f).

References

1 Li, Q. & Chen, B. Organic pollutant clustered in the plant cuticular membranes: Visualizing the distribution of phenanthrene in leaf cuticle using two-photon confocal scanning laser microscopy. *Environ. Sci. Technol.* **48**, 4774-4781 (2014).

2 Barthlott, W. *et al.* Classification and terminology of plant epicuticular waxes. *Botanical Journal of the Linnean Society* **126**, 237-260 (1998).
